# Ultimate Intrinsic SNR Depends on B_0_ Orientation

**DOI:** 10.1002/mrm.70577

**Published:** 2026-08-25

**Authors:** Yuting Wang, Markus W. May, Marcel Gratz, Mark E. Ladd, Stephan Orzada

**Affiliations:** ^1^ Medical Physics in Radiology German Cancer Research Center (DKFZ) Heidelberg Germany; ^2^ Faculty of Physics and Astronomy Heidelberg University Heidelberg Germany; ^3^ High‐Field and Hybrid MR Imaging University Hospital Essen Essen Germany; ^4^ Erwin L. Hahn Institute for MR Imaging University of Duisburg‐Essen Essen Germany; ^5^ Faculty of Medicine Heidelberg University Heidelberg Germany

**Keywords:** B_0_ orientation, in silico, MRI, patient orientation, uiSNR

## Abstract

**Purpose:**

This work aims to investigate the effect of $B_{0}$ orientation on the uiSNR in MRI.

**Methods:**

Electromagnetic simulations were performed on both realistic body models and simplified geometric models. Comparisons between (1) opposite $B_{0}$ directions along the head‐foot (HF) axis, corresponding to head‐first and feet‐first patient orientations, at various field strengths, and (2) $B_{0}$ along the HF, anterior–posterior (AP), and left–right (LR) directions at 0.55 T, corresponding to closed‐bore (HF) and open (AP/LR) MR systems. The voxel‐wise ratio of uiSNR between different $B_{0}$ orientations was calculated to characterize spatial variations throughout the models.

**Results:**

For closed‐bore MRI systems, flipping the patient orientation results in a spatial redistribution of uiSNR, and the magnitude of this redistribution increases with $B_{0}$ field strength. At 0.55 T, open MR systems with either AP‐ or LR‐oriented $B_{0}$ yield higher uiSNR than closed‐bore systems in the center of cylindrical‐shaped structures. In realistic body models, LR‐oriented $B_{0}$ yields higher uiSNR than AP‐oriented $B_{0}$ in most regions at 0.55 T, with both orientations outperforming HF‐oriented $B_{0}$ in the center of the body.

**Conclusion:**

The uiSNR distribution varies with $B_{0}$ orientation in realistic body models. At around 0.55 T, the flexibility in $B_{0}$ orientation enabled by different magnet configurations may serve as an additional degree of freedom for improving imaging performance, as uiSNR is strongly influenced by the orientation of the main field.

## 1 Introduction

SNR is a key metric of image quality in MRI, as it directly affects resolution and scan time. Ultimate intrinsic SNR (uiSNR) [1, 2, 3], defined as the maximum intrinsic SNR (only sample noise and no detector noise) achievable by any RF receive array, is a useful framework for investigating SNR because it minimizes the influence of factors such as the specific design of the receive array. While uiSNR is a function of the static magnetic field $B_{0}$ (both strength and orientation) and the imaged object [3, 4, 5, 6], existing studies have primarily focused on its dependence on $B_{0}$ strength, implicitly assuming a fixed $B_{0}$ orientation. However, the relative orientation between the patient and the $B_{0}$ field can affect the local receive field distribution, thereby influencing the corresponding uiSNR.

Although $B_{0}$ can theoretically assume arbitrary orientations, many such configurations are not physically feasible within the constraints of current MR scanner architectures. This study investigates two practically relevant scenarios involving alternative $B_{0}$ orientations as follows: (1) the effect of patient orientation relative to the $B_{0}$ field in closed‐bore systems (Figure 1A), and (2) the impact of different $B_{0}$ field directions relative to the patient anatomy, as encountered in various system configurations (Figure 1B).

**FIGURE 1:**
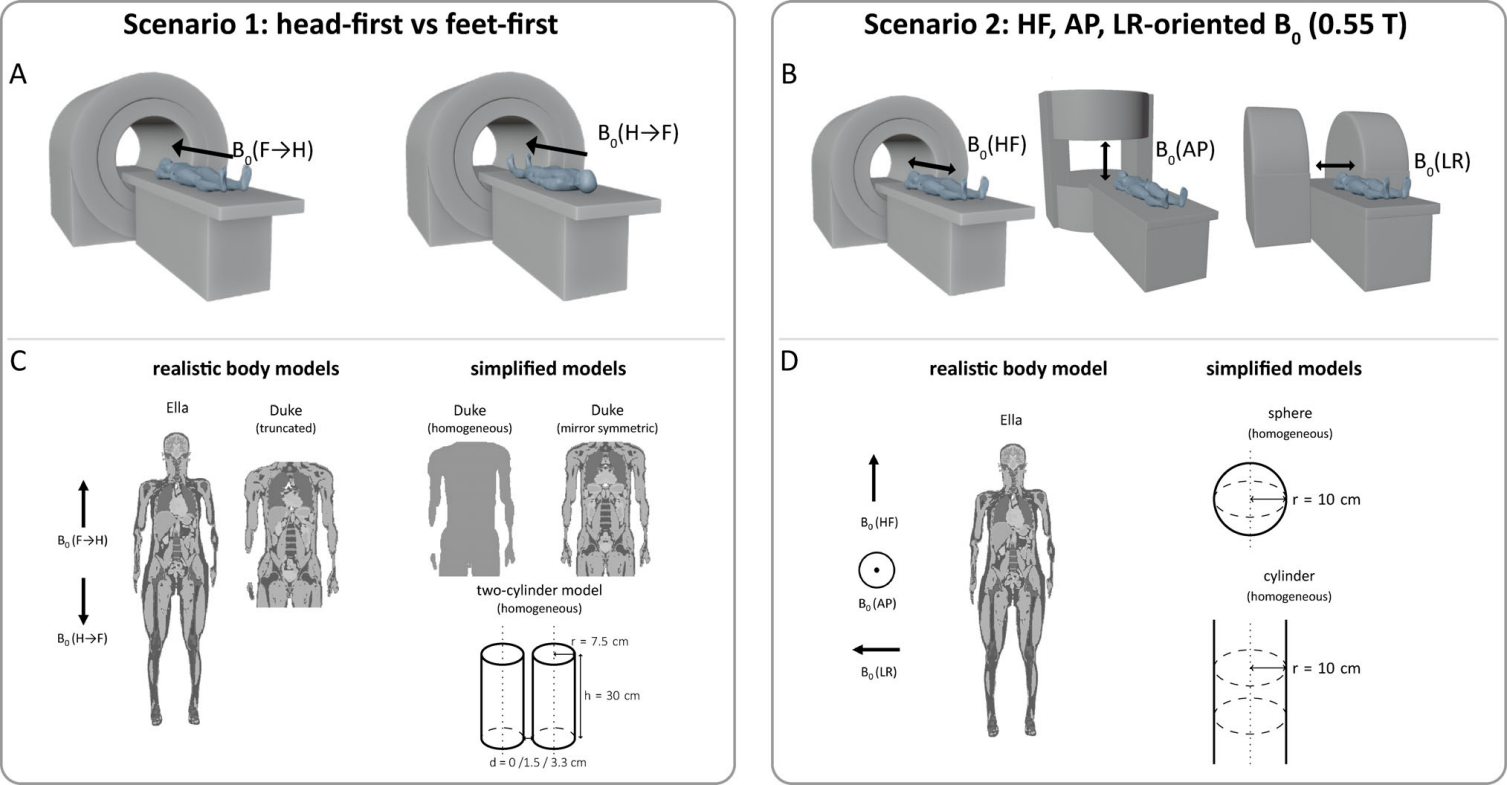
Illustration of the two investigated B_0_ orientation scenarios and their corresponding simulation setups. (A) Schematic of the first scenario: Head‐first versus feet‐first patient positioning in closed‐bore MR systems, which effectively corresponds to head‐to‐foot (H → F) or foot‐to‐head (F → H) B_0_ orientations, depending on the B_0_ polarity of the scanner. (B) Schematic of the second scenario: Three orthogonal B_0_ orientations relative to the patient anatomy. These include head‐foot (HF) oriented B_0_ for closed‐bore systems, as well as anterior–posterior (AP) and left–right (LR) oriented B_0_ for different open MR system configurations that exist at low‐ to mid‐field strengths (e.g., 0.55 T). (C) Simulation setups for the patient orientation study shown in (A), utilizing realistic body models (Ella and truncated Duke), simplified body derived from Duke (homogeneous and mirror symmetric), and a two‐cylinder model with varying separation. (D) Simulation setups for the orthogonal B_0_ orientations shown in (B), including the realistic body model Ella and two homogeneous geometric models (a sphere and a cylinder).

First, for closed‐bore MR systems, patients can be put in either head‐first or feet‐first, which corresponds to two opposite $B_{0}$ orientations (Figure 1A). In practice, patient orientation is chosen mainly depending on the body part to be scanned, so that the target anatomy is positioned in the center of the magnet and so that patient comfort is maximized. A reversal of the $B_{0}$ orientation is equivalent to an interchange between $B_{1}^{+}$ and $B_{1}^{-}$ field patterns [7]. As field strength increases, the $B_{1}^{+}$ and $B_{1}^{-}$ fields exhibit a characteristic “twisting” or wave‐like behavior due to shortened wavelengths and frequency‐dependent dielectric properties of different tissues [8, 9], implying that $B_{0}$ orientation can significantly alter RF transmission and reception profiles. For instance, studies have reported substantial variations in local SAR and the heating of implanted devices depending on the $B_{0}$ direction [10, 11, 12]. Since these effects originate from the interaction between the RF fields and the asymmetric human anatomy, it is reasonable to expect that RF reception is also influenced by the $B_{0}$ orientation. The concept of uiSNR provides a hardware‐independent perspective to investigate this effect, allowing us to focus purely on the role of the human body as an asymmetric heterogeneous scatterer rather than the characteristics of specific RF coil designs. Some early studies on uiSNR have relied on rotationally symmetric models, such as spheres or cylinders [4, 13, 14]. In these cases, when $B_{0}$ is aligned with the axis of symmetry, reversing the direction of $B_{0}$ does not change the uiSNR distribution. While recent advances in computational techniques and resources have enabled uiSNR investigations using realistic body models [5, 6, 15, 16], a fixed $B_{0}$ direction is typically assumed. Consequently, the potential dependence of uiSNR on patient orientation in realistic human anatomy has not yet been investigated.

Second, at field strengths up to 1.2 T, MR systems exist in both closed‐bore configurations ($B_{0}$ oriented along the head‐foot (HF) direction) and open configurations ($B_{0}$ along the anterior–posterior (AP) or left–right (LR) direction) (Figure 1B). This diversity in magnet design offers greater flexibility in $B_{0}$ orientation relative to the patient. However, in previous uiSNR studies, whether based on simplified geometric models or realistic body models, a fixed $B_{0}$ orientation was generally assumed, typically corresponding to that of a closed‐bore MR system. As a result, the dependence of uiSNR on these orthogonal $B_{0}$ orientations at the same field strength has not been fully explored. Since the human body exhibits significant anatomical anisotropy, the interaction between the RF field and the geometry of the human body is expected to vary across these configurations. Such an investigation is of interest to determine whether specific $B_{0}$ orientations offer a fundamental advantage for imaging particular regions of interest, potentially providing theoretical guidance for future system design.

Building upon the uiSNR simulation framework employed in our previous study [16], this work extends the investigation to evaluate the effect of $B_{0}$ orientation on uiSNR in realistic body models. To better illustrate the underlying physics, we also provide supporting evidence through simulations with simplified models as well as semi‐analytical results in geometric models where applicable. Focusing on the two practically relevant scenarios described above, we highlight a previously overlooked phenomenon that uiSNR is not a fixed limit for a given anatomy but depends fundamentally on the $B_{0}$ orientation.

## 2 Methods

This section is organized as follows: we first present models simulated in various scenarios with different $B_{0}$ orientations, followed by a description of the computational framework employed to derive the uiSNR.

### 2.1 Simulated Models and $B_{0}$ Orientation Configurations

The Duke and Ella body models from the Virtual Family [17] (IT'IS Foundation, Zürich, Switzerland) were used for the electromagnetic (EM) simulations in this work. Both models were discretized into voxel representations with a 3 mm isotropic voxel size. Frequency‐dependent dielectric properties were assigned to each tissue according to the IT'IS database [18], corresponding to the specific $B_{0}$ field strengths being simulated.

#### 2.1.1 Scenario 1: Head‐First versus Feet‐First Patient Orientation

To investigate the dependence of uiSNR on longitudinal $B_{0}$ orientation (head‐first vs. feet‐first patient orientation), both Duke and Ella models were employed for simulations at field strengths of 0.55 T, 1.5 T, 3 T, 5 T, 7 T, 10.5 T, 11.7 T, and 14 T (Figure 1C, left). To maintain computational efficiency, the Duke model was truncated from the neck to the proximal third of the femur, whereas the smaller Ella model was simulated as a complete body model. The validity of using truncated model to estimate uiSNR in the torso has been previously established [16]. To investigate the $B_{0}$ orientation dependence, uiSNR data previously simulated for a fixed orientation (head‐to‐foot, H → F) [16] were reused and compared with new results obtained for the reversed $B_{0}$ orientation (foot‐to‐head, F → H).

To better understand the characteristics of the observed uiSNR variations, several simplified models were simulated at 7 T with both H → F‐oriented and F → H‐oriented $B_{0}$ (Figure 1C, right). Specifically, a homogeneous body model with average body tissue properties at 7 T ($\epsilon_{r}^{\prime }$ = 42.49, σ = 0.57 S·m^−1^) and a mirror‐symmetric body model derived by mirroring the left half of the Duke model were used to examine how internal tissue heterogeneity and structural asymmetry relate to the uiSNR differences induced by $B_{0}$ reversal. Additionally, a two‐cylinder model with separations of 0, 1.5, and 3.3 cm was simulated using average body tissue properties at 7 T ($\epsilon_{r}^{\prime }$ = 42.49, σ = 0.57 S·m^−1^). This setup represents one of the simplest geometric configurations in which the uiSNR dependence on $B_{0}$ reversal remains observable. The two‐cylinder model was discretized with a 3 mm isotropic voxel size for numerical simulation, as the inter‐cylinder coupling makes an analytical approach impractical.

#### 2.1.2 Scenario 2: Orthogonal $B_{0}$ Orientations at 0.55 T

The Ella model was used to investigate how uiSNR in a human body varies across three orthogonal $B_{0}$ orientations at 0.55 T: HF‐oriented $B_{0}$ in closed‐bore systems, and AP‐ and LR‐oriented $B_{0}$ in open systems (Figure 1D, left). To provide an idealized reference, a sphere model (*r* = 0.1 m) and an infinitely long cylinder model (*r* = 0.1 m) were simulated using average brain tissue properties ($\epsilon_{r}^{\prime }$ = 148.09, σ = 0.30 S·m^−1^) and average body tissue properties ($\epsilon_{r}^{\prime }$ = 76.77, σ = 0.38 S·m^−1^), respectively (Figure 1D, right). The dimensions of these models were chosen to be comparable to the scales of the human head and thigh. For the homogeneous cylinder, its rotational symmetry implies that a distinction is only necessary between $B_{0}$ oriented parallel to the axis (HF‐oriented $B_{0}$ in Figure 1D) and $B_{0}$ oriented perpendicular to the axis. Consequently, the uiSNR maps for AP‐ and LR‐oriented $B_{0}$ can be obtained via a 90° rotation, making rotated simulations unnecessary. For the homogeneous sphere, the uiSNR map for any arbitrary $B_{0}$ direction can be derived by rotating the map generated for a fixed $B_{0}$ orientation (e.g., HF‐oriented $B_{0}$ in Figure 1D). It is important to note that while the sphere itself is spherically symmetric, the resulting uiSNR distribution does not possess spherical symmetry because the chosen $B_{0}$ direction breaks the original symmetry of the geometry. For instance, prior studies have reported that uiSNR profiles along radii parallel and perpendicular to $B_{0}$ are distinct [3]. Our work expands upon this observation and provides a further interpretation of this phenomenon in the context of different scanner designs. Semi‐analytical methods were employed for these idealized geometries to validate the trends observed in the more complex numerical simulations of the Ella model.

### 2.2 uiSNR Calculation

The calculation of uiSNR was performed in two sequential stages: the construction of an EM basis set, followed by the voxel‐wise calculation of uiSNR based on these bases.

#### 2.2.1 Construction of EM Basis Sets

For realistic body models and the two‐cylinder model, where analytical mode expansions are not feasible, we employed the numerical approach proposed by Guerin et al. [5], using the volume integral solver MARIE [19, 20, 21] to construct EM basis sets. The simulations followed the methodology employed in our previous work [16], which provides a detailed illustration of the setup along with discussions on convergence analysis and body model truncation. For completeness, a brief overview is provided here: for each model, a voxelized dipole cloud conformal to the model surface and located 3 cm away was constructed. A set of EM bases was generated by randomly exciting this dipole cloud. As the number of bases increases, the set progressively approximates a complete basis set, allowing for asymptotic estimation of uiSNR. In this work, EM basis sets consisting of 3000 basis vectors ${\left\{({\vec{E}}_{i}\left(\vec{r}\right),\;{\vec{B}}_{1,i}\left(\vec{r}\right))\right\}}_{i=1}^{3000}$ were calculated for each simulation. This number was chosen based on our previous validation [16], which demonstrated that 3000 bases provide sufficient convergence for the deeper‐lying regions of interest within the torso, while maintaining a manageable computational load.

In parallel, for the spherical and infinitely long cylindrical models, we performed mode expansions using vector wave functions to provide analytical EM basis sets (see Appendix A) [3, 4, 13, 22, 23]. To ensure sufficient numerical convergence, the expansion order was set to $l_{\max}$ = 70 for the spherical model, while the ranges n = −40 … 40 and h = −50 … 50 were adopted for the cylinder model, following Lattanzi et al. [4].

#### 2.2.2 Orientation‐Dependent $B_{1}^{-}$ and uiSNR Calculation

The RF receive field, $B_{1}^{-}$, is a primary determinant of uiSNR, and its calculation is intrinsically linked to the orientation of $B_{0}$. For any given $B_{0}$ orientation, a local right‐handed coordinate frame $\left({\hat{v}}_{1},{\hat{v}}_{2},{\hat{v}}_{3}\right)$ can be constructed at each point. In this frame, ${\hat{v}}_{3}$ is defined as the unit vector aligned with the direction of $B_{0}$, while ${\hat{v}}_{1}$ and ${\hat{v}}_{2}$ are two mutually perpendicular unit vectors spanning the local transverse plane. The numerical simulations based on random dipole clouds were performed in a Cartesian coordinate system, whereas the mode expansions for the geometric models were performed in spherical or cylindrical coordinates. Corresponding rotation matrices were used to transform the simulation coordinate system into the constructed coordinate frame aligned with $B_{0}$, which can also be used to calculate components of ${\vec{B}}_{1}$ in the new coordinate frame (Appendix B). This point‐wise frame transformation ensures that the RF fields are correctly decomposed into different rotating components relative to the direction of $B_{0}$. According to the principle of reciprocity [24], when ${\hat{v}}_{3}$ is aligned with the direction of $B_{0}$, $B_{1}^{-}$ can be calculated as [7]:

$$B_{1}^{-}\left(\vec{r}\right)=\frac{1}{2}\left(B_{1}{\left(\vec{r}\right)}_{v_{1}}+iB_{1}{\left(\vec{r}\right)}_{v_{2}}\right).$$

With the corresponding EM basis set and assuming a homogeneous 90° flip angle excitation, the uiSNR in the unit of $\frac{\sqrt{\mathrm{Hz}}}{\mathrm{mL}}$ can be calculated voxel‐wise as [3, 25]:

$$\text{uiSNR}\left(\vec{r}\right)=\frac{2{\omega M}_{0}\left(\vec{r}\right)}{\sqrt{4k_{b}T}}\sqrt{{B_{1}^{-}}^{H}\left(\vec{r}\right)\Psi^{-1}B_{1}^{-}\left(\vec{r}\right)},$$

where $\Psi_{\mathrm{ij}}=\int_{V}\sigma \left({\vec{r}}^{\prime }\right){\vec{E}}_{i}\left({\vec{r}}^{\prime }\right){\vec{E}}_{j}^{H}\left({\vec{r}}^{\prime }\right)d^{3}{\vec{r}}^{\prime }$ is the noise covariance matrix, $B_{1}^{-}\left(\vec{r}\right)$ is the tensor of RF receive fields constructed by concatenating the 3000 individual RF receive fields $B_{1}^{-}\left(\vec{r}\right)$, ω is the Larmor frequency, $M_{0}$ is the equilibrium magnetization, $k_{b}$ is Boltzmann's constant, and T is the absolute temperature.

For each model configuration, the ratio of uiSNR values between different $B_{0}$ orientations was evaluated to identify preferred orientations. The deviation of this ratio from unity is of interest, as it indicates the SNR gain or loss associated with a specific $B_{0}$ orientation, thereby identifying a better configuration.

### 2.3 Computational Environment

EM bases construction and uiSNR calculation were conducted in MATLAB R2023b (MathWorks, Natick, MA, USA) on a server equipped with Ubuntu 24.04 LTS featuring 1.5 TB RAM, dual AMD Epyc 9654 processors (each with 96 cores/192 threads), and two NVIDIA H100 GPUs (each with 80 GB memory).

## 3 Results

### 3.1 Scenario 1: Head‐First versus Feet‐First Patient Orientation

Figure 2 shows that the uiSNR ratio maps vary when the $B_{0}$ orientation is reversed, and that the magnitude of these variations increases with $B_{0}$ field strength. As illustrated in the liver, the uiSNR ratio remains close to 1 across all investigated field strengths, indicating that the interior of relatively uniform tissues is insensitive to the choice of patient orientation. However, localized variations occur near regions with high dielectric property contrast, for example at anatomical boundaries.

**FIGURE 2:**
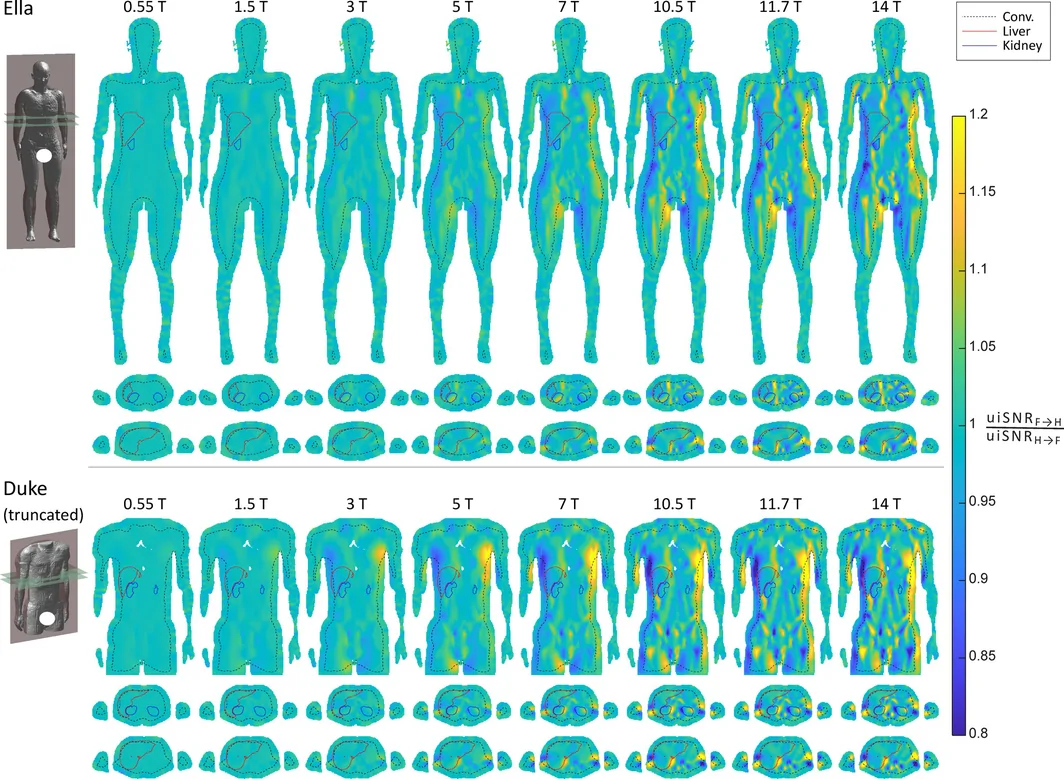
The uiSNR ratio maps for F → H versus H → F B_0_ orientations in the Ella and truncated Duke models across a B_0_ strength range from 0.55 T to 14 T, displayed in a coronal slice and two transverse slices, respectively. The dashed lines mark the boundary of convergence, defined as a uiSNR difference of less than 1% between uiSNR calculations using 2900 and 3000 EM bases. Liver and kidneys are contoured for anatomical reference.

This trend is also evident in the statistical distributions of the ratio values (Figure 3), where the simulated realistic body models were categorized into three bulk regions of interest: the truncated Duke torso, the Ella torso covering the same anatomical extent (neck to the proximal third of the femur), and the Ella head (above the neck). At lower field strengths, the ratio values cluster around 1, while the distribution becomes broader as the field strength increases. At 14 T, the ratio values span from 0.66 to 1.60 in the torso of the Duke model. This indicates that, at certain locations, a favorable $B_{0}$ orientation can yield up to 50% higher uiSNR than an unfavorable orientation. Furthermore, the deviation becomes larger for models with greater geometric dimensions (Duke torso > Ella torso > Ella head), suggesting that the effect is related to RF wave behavior. For visualization of the statistical distributions, a logarithmic scale was used for the ratio axis to avoid visual bias arising from whether F → H or H → F is used as the numerator in the ratio calculation. After this correction, the ratio distributions in these bulk regions of interest exhibit a highly symmetric pattern around 1, indicating that there is no overall preference for either $B_{0}$ orientation despite substantial local variations.

**FIGURE 3:**
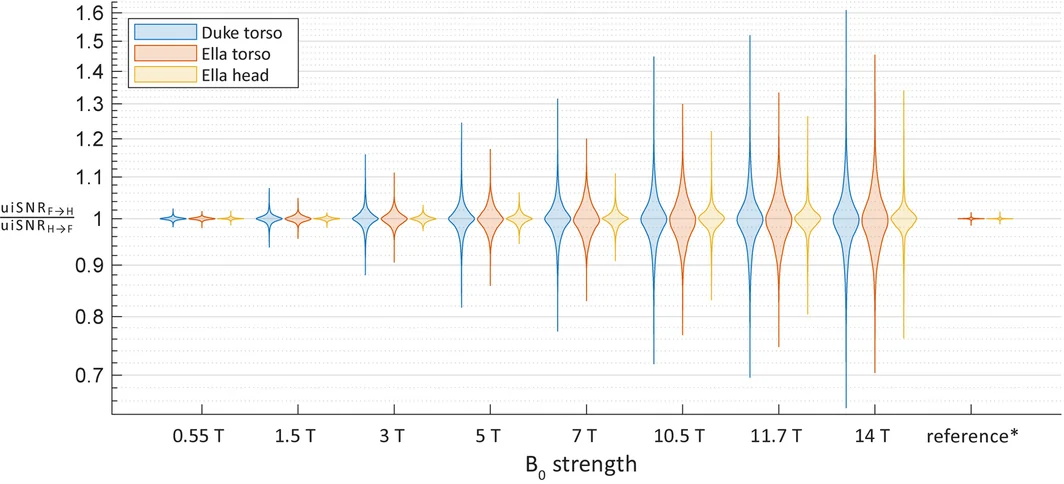
Violin plots of uiSNR ratio distributions (F → H vs. H → F B_0_ orientations) for the Duke torso, Ella torso, and Ella head. Only voxels located within the convergence boundary are included in the analysis. The distributions are normalized by width. The y‐axis is on a logarithmic scale to ensure that the distribution shapes remain visually unbiased regardless of the arbitrary choice of the numerator and denominator (F → H vs. H → F or H → F vs. F → H). *As a reference, the 7 T simulation in the Ella model was performed twice using the same B_0_ orientation to illustrate the baseline variation caused by the randomness of the EM basis set.

Simplified models including a homogeneous Duke model, a mirror‐symmetric Duke model, and two‐cylinder models with varying separations were employed to illustrate several properties of this phenomenon (Figure 4), including spatial scale and impact of mirror symmetry. Comparison of the anatomical and homogeneous Duke models (Figure 4A,B) demonstrates that internal tissue structure leads to uiSNR variations on a smaller spatial scale, whereas in the homogeneous model the directional preference is primarily determined by the overall shape. In the mirror‐symmetric Duke model (Figure 4C), regions with opposite $B_{0}$ orientation preference in terms of uiSNR occur symmetrically in pairs. On the plane of symmetry, corresponding to the central sagittal plane of the mirror‐symmetric Duke model, the uiSNR ratio remains close to 1 throughout, reflecting the absence of directional preference. By comparison, in the original Duke model, the uiSNR ratio in the central sagittal plane deviates from 1 in certain regions due to the intrinsic asymmetry of human anatomy.

**FIGURE 4:**
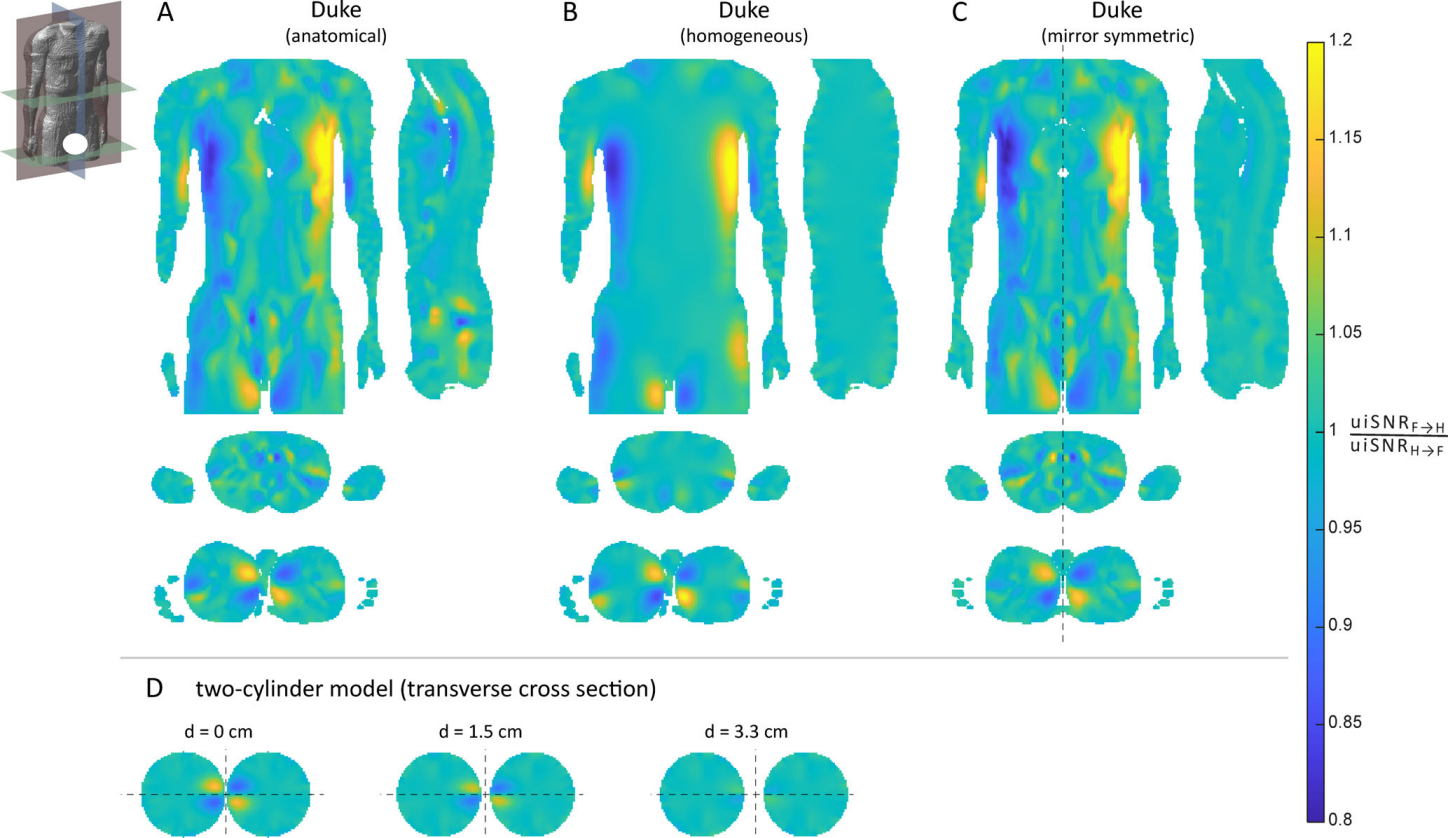
The uiSNR ratio maps for F → H versus H → F B_0_ orientations in simplified models at 7 T, including: (A) the original Duke model as a reference, (B) a homogeneous Duke model, (C) a mirror symmetric Duke model, and (D) two‐cylinder models with varying separations. Dashed lines indicate the planes of mirror symmetry where applicable.

The two‐cylinder model was introduced as a simple geometry that we found useful for illustrating this phenomenon (Figure 4D). In the two‐cylinder models with varying separations, the transverse uiSNR profile varies only weakly along the longitudinal direction away from the cylinder ends. Therefore, only the central transverse slice is shown for each separation distance. These models exhibit distinct paired regions favoring opposite $B_{0}$ orientations in terms of uiSNR, primarily in the regions facing the neighboring cylinder. The ratio pattern also exhibits a four‐quadrant structure, in which diagonally opposite regions show similar behavior while adjacent regions show opposite preference. The effect becomes more pronounced when the cylinders are closer together. The two‐cylinder models possess two orthogonal planes of mirror symmetry aligned with $B_{0}$, while only one of these planes intersects the cylinders. On this intersecting symmetry plane, the uiSNR ratio remains close to 1. The two‐cylinder geometry also roughly mimics the structure of the thighs, where a similar ratio pattern can be observed.

### 3.2 Scenario 2: Orthogonal $B_{0}$ Orientations at 0.55 T

Figure 5A,B compare the uiSNR ratio between AP‐ and HF‐oriented $B_{0}$ and between LR‐ and HF‐oriented $B_{0}$ in the Ella model at 0.55 T, respectively. $B_{0}$ oriented perpendicular to the HF direction (i.e., AP‐ or LR‐oriented $B_{0}$) yields higher uiSNR in the central regions of cylindrically shaped structures with their main axis approximately aligned with the HF direction, such as the torso (approximately 10% higher) and legs (approximately 15% higher). A similar trend can be observed in the foot, which can be idealized as an elliptic cylinder oriented perpendicular to the leg. In this case, HF‐oriented $B_{0}$ yields higher uiSNR than AP‐oriented $B_{0}$, consistent with the higher uiSNR observed when $B_{0}$ is oriented perpendicular to the cylinder axis. This trend is further supported by the infinite‐cylinder model with a radius of 10 cm (Figure 5E), which mimics cylindrically shaped anatomical structures such as the legs. Semi‐analytical uiSNR calculations based on mode expansions confirm that a $B_{0}$ orientation perpendicular to the cylinder axis yields about 16% higher uiSNR at the cylinder axis than a $B_{0}$ orientation parallel to the axis.

**FIGURE 5:**
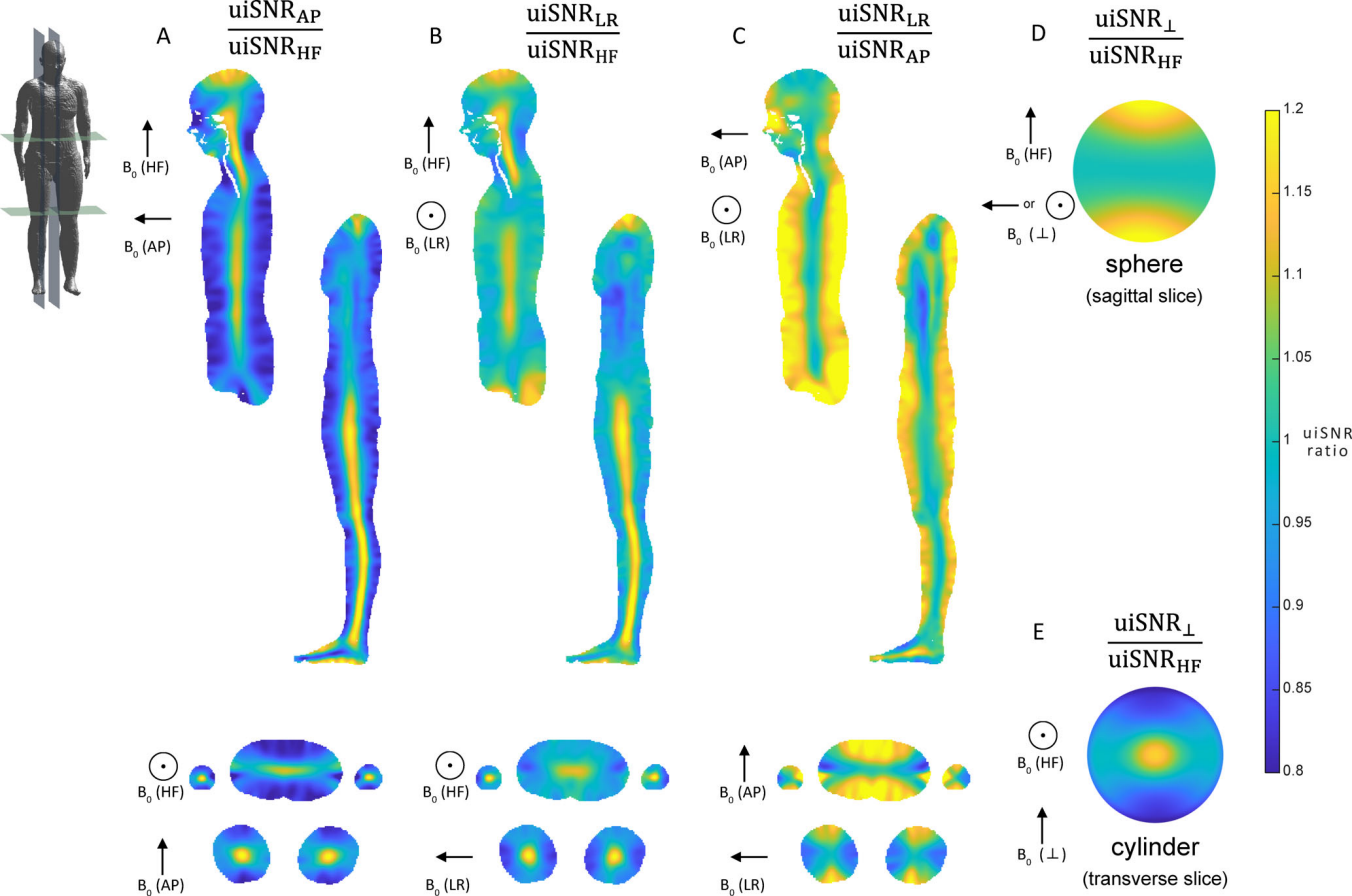
Two sagittal and two transverse slices of the uiSNR ratio maps in the Ella model comparing (A) AP and HF, (B) LR and HF, and (C) LR and AP B_0_ orientations at 0.55 T. (D) Sagittal cross‐section of the uiSNR ratio map in a homogeneous sphere comparing B_0_ perpendicular to the HF direction with B_0_ along the HF direction at 0.55 T. (E) Transverse cross section of the uiSNR ratio map in an infinitely long homogeneous cylinder comparing B_0_ perpendicular to the HF direction with B_0_ along the HF direction at 0.55 T.

In peripheral regions, particularly near surfaces with normals parallel to AP‐ or LR‐oriented $B_{0}$, HF‐oriented $B_{0}$ yields higher uiSNR, as reflected by the darker blue regions in Figure 5A,B. Similar behavior is observed in the cylinder model (Figure 5E). The cylindrical symmetry of uiSNR is disrupted when $B_{0}$ is oriented perpendicular to the cylinder axis, leading to higher uiSNR near surfaces tangent to the $B_{0}$ direction than near surfaces perpendicular to it. Due to the geometry of the human body, LR‐oriented $B_{0}$ produces higher uiSNR than AP‐oriented $B_{0}$ in most regions. This relative advantage of the LR orientation is also reflected in the LR‐to‐AP uiSNR ratio map (Figure 5C).

Another observation can be made at the top of the head in the realistic body model, where AP‐ or LR‐oriented $B_{0}$ yields higher uiSNR, consistent with the result obtained in the homogeneous sphere model with a radius 10 cm (Figure 5D), which mimics the approximately spherical geometry of the head.

Figure 6 shows violin plots of the voxel‐wise uiSNR ratio distributions between the three orthogonal $B_{0}$ orientations. The statistical distribution of uiSNR ratio between AP‐ and HF‐oriented $B_{0}$ is skewed toward values smaller than 1, indicating that more voxels achieve higher uiSNR with HF‐oriented $B_{0}$. In contrast, the distribution of uiSNR ratio between LR‐ and HF‐oriented $B_{0}$ is centered around 1, and each orientation yields higher uiSNR in roughly half of the voxels. Although open MR configurations with either AP‐ or LR‐oriented $B_{0}$ do not exhibit an overall statistical advantage over HF‐oriented $B_{0}$, the spatial distributions shown in Figure 5 indicate that voxels favoring AP‐ or LR‐oriented $B_{0}$ are predominantly located in central regions. This observation is noteworthy because the central regions intrinsically exhibit lower uiSNR [5, 6, 16]. Compared with AP‐oriented $B_{0}$, LR‐oriented $B_{0}$ yields higher uiSNR in approximately 70% of the voxels, suggesting a relative uiSNR advantage of LR‐oriented $B_{0}$.

**FIGURE 6:**
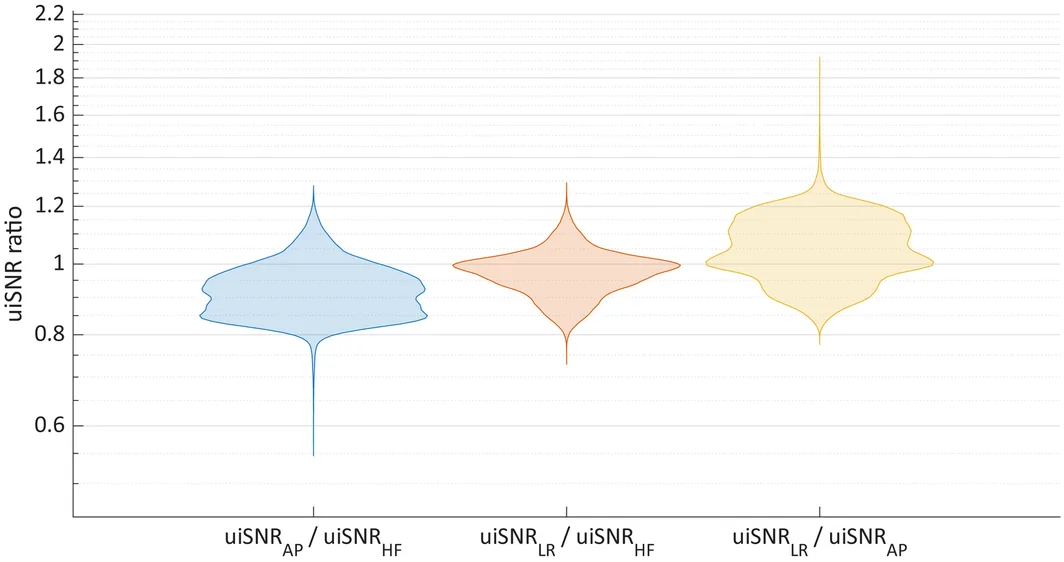
Violin plots of the uiSNR ratio distributions between the three orthogonal B_0_ orientations (HF, AP, and LR) in the Ella model at 0.55 T. The distributions are normalized by width. The y‐axis is shown on a logarithmic scale to ensure that the distribution shapes remain visually unbiased with respect to the arbitrary choice of numerator and denominator.

## 4 Discussion

### 4.1 Limitations of uiSNR Simulations in Realistic Body Models

The uiSNR simulations in this work followed the methodology proposed by Guerin et al. [5] and reused part of the data of our previous work on $B_{0}$ field strength dependence of uiSNR [16], in which the effect of truncation of body model, and the convergence behavior of the EM basis set with 3000 basis vectors were investigated. As the present study focuses on the qualitative dependence of uiSNR on $B_{0}$ orientation rather than on technical aspects of electromagnetic simulations, more advanced techniques aimed at improving numerical accuracy and computational efficiency, including piecewise‐linear basis support [26], reduced‐order modeling [27, 28] and tensor decomposition for volume integral equations [29, 30], were not employed, as they were not essential for the qualitative comparisons presented in this work. Alternative basis constructions, such as the Rao‐Wilton‐Glisson (RWG) [31]‐based framework proposed for estimation of ideal current patterns in realistic body models [15], may provide additional insight into coil design for different $B_{0}$ orientations, but were beyond the scope of the present study.

Due to the randomness and limited completeness of the employed basis sets, uiSNR values computed near the surface are numerically unstable. This behavior can be observed as irregular stripe‐like patterns in the peripheral regions of the ratio maps (Figures 2 and 5). Although the peripheral region is also where some of the reported effects are observed, we argue below that these effects are not artifacts arising from numerical randomness or incomplete convergence of the basis set.

For Scenario 1, the convergence boundary was defined in Figure 2 according to the criterion adopted in our previous work [16], where convergence was considered achieved when the uiSNR difference between calculations using 2900 and 3000 EM basis vectors was less than 1%. To estimate the baseline variation caused by the randomness of the EM basis set, the simulation of the Ella model at 7 T was repeated using the same $B_{0}$ orientation. As shown in Figure 3, the resulting variation within the defined convergence boundary was small, supporting the adopted convergence criterion. Moreover, the observed variation was substantially smaller than the orientation‐dependent uiSNR differences, indicating that the reported effect is unlikely to be dominated by numerical variability.

For Scenario 2, the key observation is that open MR configurations with AP‐ or LR‐oriented $B_{0}$ provide higher uiSNR than HF‐oriented $B_{0}$ in the central regions of cylindrically shaped body parts. Based on the previous convergence analysis, convergence is considered achieved for HF‐oriented $B_{0}$ in these regions. For AP‐ and LR‐oriented $B_{0}$, the uiSNR obtained using 3000 EM basis vectors represents a lower‐bound estimate of the theoretical uiSNR, as the finite basis set only provides an approximation to the complete electromagnetic basis. Consequently, the reported uiSNR ratios relative to HF‐oriented $B_{0}$ are more likely to underestimate than overestimate the orientation‐dependent differences. Furthermore, uiSNR calculations in spherical and cylindrical models were performed using an independent methodology, and similar, although less noisy, spatial patterns were observed. These results provide indirect support for the effects observed in the simulations of realistic body models.

### 4.2 Scenario 1: Head‐First versus Feet‐First Patient Orientation

The observed differences between head‐first and feet‐first orientations arise from the electromagnetic properties of the RF fields, including polarization directionality and wavelength effects.

The RF receive field $B_{1}^{-}$ and the transmit field $B_{1}^{+}$ correspond to different circularly polarized components of the magnetic field and therefore possess inherent directionality. This property gives rise to asymmetric spatial distributions of $B_{1}^{+}$ and $B_{1}^{-}$ of RF coils. Since reversing the $B_{0}$ orientation is equivalent to interchanging $B_{1}^{+}$ and $B_{1}^{-}$ [7], orientation‐dependent behaviors have been reported for specific coil elements [32]. However, the effect illustrated in this work differs from these coil‐dependent $B_{1}^{+}$/$B_{1}^{-}$ asymmetries, because uiSNR is independent of any particular receive coil design. As uiSNR is computed voxel‐wise, each spatial location is associated with its own optimal electromagnetic field. Our results therefore reveal a more intrinsic effect: a spatially varying RF polarization preference arising from the heterogeneous structure of the human body itself.

The stronger orientation dependence observed at higher $B_{0}$ is consistent with the increasing asymmetry between $B_{1}^{+}$ and $B_{1}^{-}$ distributions reported at higher RF frequencies [7, 8]. Such asymmetries become more pronounced as the electrical size of the object increases and the RF fields depart from the quasistatic regime, allowing $B_{0}$ orientation dependence of uiSNR to emerge. This interpretation is further supported by the comparison between realistic body models of different sizes (Duke torso, Ella torso, and Ella head). However, the comparison between anatomical and homogeneous body models suggests that electrical size alone cannot explain the observed spatial patterns. Instead, the spatial scale of the observed effect is comparable to that of the underlying anatomical structures, suggesting that the observed patterns are closely related to local geometry. Together, these observations suggest that the effect reflects a complex interaction between RF wave behavior and anatomical structure.

In a more realistic setting, it would be valuable to consider both the orientation dependence of receive coil elements and the intrinsic anatomy‐driven electromagnetic response identified in this work. Simulations incorporating both realistic body models and realistic coil designs could help elucidate how these two effects interact and identify applications in which patient‐orientation effects become significant.

RF transmission and reception are intrinsically linked, representing complementary aspects of the same physical process. If a location within the body favors a particular orientation for signal reception due to the interplay between global and local geometries, a corresponding orientation dependence may also be expected during excitation, which is likewise influenced by the direction of $B_{0}$. Therefore, patient‐orientation effects warrant further investigation in the context of RF transmission, particularly with respect to transmit‐field distributions and SAR [10, 11, 12].

### 4.3 Scenario 2: Orthogonal $B_{0}$ Orientations at 0.55 T

The differences between different $B_{0}$ orientations originate from the distinct directional components of the magnetic field. Taking a point at the axis of an infinitely long cylinder as an example, different $B_{0}$ orientations correspond to different sets of nonzero electromagnetic modes (see Appendix A for the definitions of the cylindrical vector wave functions used as basis functions in the expansion). When $B_{0}$ is parallel to the cylinder axis, only $M_{n,h}$ and $N_{n,h}$ with n=1 are nonzero. In contrast, when $B_{0}$ is perpendicular to the axis, $M_{n,h}$ is nonzero for n=±1, while $N_{n,h}$ is nonzero for n=0,±1. Therefore, different $B_{0}$ orientations involve different sets of modes contributing to the uiSNR. Although a similar mode expansion analysis is not feasible for realistic body models, this example illustrates that changing the orientation of $B_{0}$ alters the magnetic field components perpendicular to $B_{0}$ and therefore changes the electromagnetic field patterns contributing to uiSNR.

The improved uiSNR observed in the central regions of cylinder‐like structures, where the absolute SNR is typically lowest, suggests that open MR configurations may provide an intrinsic SNR advantage for deeper‐lying tissues. Although LR‐oriented $B_{0}$ exhibits higher uiSNR than AP‐oriented $B_{0}$, this advantage is observed predominantly in peripheral regions (Figure 5C). Previous work has shown that finite RF receive arrays approach the uiSNR much more rapidly in central regions than near the surface, where substantial deviations from ultimate performance remain even for large numbers of array elements [33]. Therefore, it remains unclear whether the relative uiSNR advantage of LR‐oriented $B_{0}$ would be preserved in practical RF coil arrays, and more realistic simulations incorporating finite receive arrays will be required to address this question.

It should be noted that the above discussion is based on uiSNR, which neglects coil noise. This limitation is particularly relevant in the mid‐field regime, where coil noise is not negligible. Nevertheless, the concept of uiSNR remains informative, as coil noise can be reduced through technical improvements, such as coil configuration optimization, material selection, and cryogenic cooling, whereas the intrinsic noise originating from the sample cannot be eliminated.

## 5 Conclusion

In this work, we demonstrated that $B_{0}$ orientation influences the uiSNR in realistic human body models. For closed‐bore MRI systems, patient orientation‐induced spatial redistribution of uiSNR becomes more pronounced at ultra‐high fields, suggesting that patient orientation should be considered in RF coil design and validation. At mid‐field strengths (around 0.55 T), LR‐oriented $B_{0}$ provides more favorable uiSNR distribution than AP‐ or HF‐oriented $B_{0}$, and the flexibility in $B_{0}$ orientation enabled by magnet design may serve as a degree of freedom for improving imaging performance.

## Funding

This work was supported by HORIZON EUROPE Framework Programme, 101078393/MRITwins.

## Conflicts of Interest

The authors declare no conflicts of interest.

## Data Availability

The data that support the findings of this study are available from the corresponding author upon reasonable request.

## Appendix A Spherical and Cylindrical Vector Wave Functions

Vector wave functions are solutions of the vector wave equation $\nabla \times \nabla \times F-k^{2}F=0$ in a certain coordinate frame [4, 13, 22].

The spherical vector wave functions are:

$$M_{l,m}\left(r,k\right)=j_{l}\left(\mathrm{kr}\right)X_{l,m}\left(\theta ,\varphi \right),$$

$$N_{l,m}\left(r,k\right)=\frac{1}{k}\nabla \times M_{l,m}\left(r,k\right),$$

where k is the complex wave number, $j_{l}$ is the spherical Bessel function of order l, and $X_{l,m}$ is the vector spherical harmonic of order (l,m).

The cylindrical vector wave functions are as follows:

$$M_{n,h}\left(r,\xi \right)=\nabla \times \left(J_{n}\left(\xi \rho \right)e^{\mathrm{in\varphi}}e^{i\frac{2\pi }{L}\mathrm{hz}}\;\hat{z}\right)=\left(i\frac{n}{\rho }\;J_{n}\left(\xi \rho \right)\hat{\rho }-\xi J_{n}^{\prime }\left(\xi \rho \right)\hat{\varphi }\right)e^{\mathrm{in\varphi}}e^{i\frac{2\pi }{L}\mathrm{hz}},$$

$N_{n,h}\left(r,\xi \right)=\frac{1}{k}\nabla \times M_{n,h}\left(r,\xi \right)=\frac{1}{k}(i\frac{2\pi }{L}h\xi J_{n}^{\prime }\left(\xi \rho \right)\hat{\rho }-\frac{2\pi }{L}\frac{\mathrm{nh}}{\rho }J_{n}\left(\xi \rho \right)\hat{\varphi }+\xi^{2}J_{n}\left(\xi \rho \right)\;\hat{z})e^{\mathrm{in\varphi}}e^{i\frac{2\pi }{L}\mathrm{hz}}.$

where L is the length of field of view and is taken as 1 m, $\xi =\sqrt{k^{2}-{\left(\frac{2\pi }{L}h\right)}^{2}}$ is the transverse wave number, $J_{n}$ and $J_{n}^{\prime }$ are the Bessel function of order n and its derivative.

## Appendix B Rotation Matrices in Different Coordinate Frames

In Cartesian coordinates (x,y,z), rotation of $B_{0}$ about the x‐axis by angle α can be expressed as

$$\left[\begin{array}{l} {\hat{v}}_{1}\\ {\hat{v}}_{2}\\ {\hat{v}}_{3} \end{array}\right]=\left[\begin{array}{lll} 1 & 0 & 0\\ 0 & \cos\alpha & -\sin\alpha \\ 0 & \sin\alpha & \cos\alpha \end{array}\right]\left[\begin{array}{l} \hat{x}\\ \hat{y}\\ \hat{z} \end{array}\right],$$

rotation about y‐axis by angle α can be expressed as

$$\left[\begin{array}{l} {\hat{v}}_{1}\\ {\hat{v}}_{2}\\ {\hat{v}}_{3} \end{array}\right]=\left[\begin{array}{lll} \cos\alpha & 0 & -\sin\alpha \\ 0 & 1 & 0\\ \sin\alpha & 0 & \cos\alpha \end{array}\right]\left[\begin{array}{l} \hat{x}\\ \hat{y}\\ \hat{z} \end{array}\right].$$

For the homogeneous sphere and cylinder model, the choice of rotation axis can be arbitrary because of symmetry.

In spherical coordinates (r,θ,φ), rotation of $B_{0}$ about $\left(\theta =\frac{\pi }{2},\varphi =\frac{\pi }{2}\right)$ by angle α can be expressed as

$$\left[\begin{array}{l} {\hat{v}}_{1}\\ {\hat{v}}_{2}\\ {\hat{v}}_{3} \end{array}\right]=\left[\begin{array}{lll} \cos\alpha \sin\theta \cos\varphi -\sin\alpha \cos\theta & \cos\alpha \cos\theta \cos\varphi +\sin\alpha \sin\theta & -\cos\alpha \sin\varphi \\ \sin\theta \sin\varphi & \cos\theta \sin\varphi & \cos\varphi \\ \sin\alpha \sin\theta \cos\varphi +\cos\alpha \cos\theta & \sin\alpha \cos\theta \cos\varphi -\cos\alpha \sin\theta & -\sin\alpha \sin\varphi \end{array}\right]\left[\begin{array}{l} \hat{r}\\ \hat{\theta }\\ \hat{\varphi } \end{array}\right].$$

In cylindrical coordinates (ρ,φ,z), rotation of $B_{0}$ about $\left(\varphi =\frac{\pi }{2},z=0\right)$ by angle α can be expressed as

$$\left[\begin{array}{l} {\hat{v}}_{1}\\ {\hat{v}}_{2}\\ {\hat{v}}_{3} \end{array}\right]=\left[\begin{array}{lll} \cos\alpha \cos\varphi & -\cos\alpha \sin\varphi & -\sin\alpha \\ \sin\varphi & \cos\varphi & 0\\ \sin\alpha \cos\varphi & -\sin\alpha \sin\varphi & \cos\alpha \end{array}\right]\left[\begin{array}{l} \hat{\rho }\\ \hat{\varphi }\\ \hat{z} \end{array}\right].$$

Flipping from the head‐first to the feet‐first configuration can be interpreted as a rotation by angle α=π. Converting from HF‐oriented $B_{0}$ to the transverse $B_{0}$ configuration can be interpreted as a rotation by angle $\alpha =\frac{\pi }{2}$.
